# Non-motor predictors of 36-month quality of life after subthalamic stimulation in Parkinson disease

**DOI:** 10.1038/s41531-021-00174-x

**Published:** 2021-06-08

**Authors:** Stefanie T. Jost, Veerle Visser-Vandewalle, Alexandra Rizos, Philipp A. Loehrer, Monty Silverdale, Julian Evans, Michael Samuel, Jan Niklas Petry-Schmelzer, Anna Sauerbier, Alexandra Gronostay, Michael T. Barbe, Gereon R. Fink, Keyoumars Ashkan, Angelo Antonini, Pablo Martinez-Martin, K. Ray Chaudhuri, Lars Timmermann, Haidar S. Dafsari, EUROPAR and the International Parkinson and Movement Disorders Society Non-Motor Parkinson’s Disease Study Group, Roongroj Bhidayasiri, Cristian Falup-Pecurariu, Beomseok Jeon, Valentina Leta, Per Borghammer, Per Odin, Anette Schrag, Alexander Storch, Mayela Rodriguez Violante, Daniel Weintraub, Charles Adler, Paolo Barone, David J. Brooks, Richard Brown, Marc Cantillon, Camille Carroll, Miguel Coelho, Tove Henriksen, Michele Hu, Peter Jenner, Milica Kramberger, Padma Kumar, Mónica Kurtis, Simon Lewis, Irene Litvan, Kelly Lyons, Davide Martino, Mario Masellis, Hideki Mochizuki, James F. Morley, Melissa Nirenberg, Javier Pagonabarraga, Jalesh Panicker, Nicola Pavese, Eero Pekkonen, Ron Postuma, Raymond Rosales, Anthony Schapira, Tanya Simuni, Fabrizio Stocchi, Indu Subramanian, Michele Tagliati, Michele Tinazzi, Jon Toledo, Yoshio Tsuboi, Richard Walker

**Affiliations:** 1 University of Cologne, Faculty of Medicine and University Hospital Cologne, Department of Neurology, Cologne, Germany; 2 University of Cologne, Faculty of Medicine and University Hospital Cologne, Department of Stereotaxy and Functional Neurosurgery, Cologne, Germany; 3 Parkinson Foundation International Centre of Excellence, King’s College Hospital, London, UK; 4 Department of Neurology, University Hospital Giessen and Marburg, Campus Marburg, Marburg, Germany; 5 Department of Neurology and Neurosurgery, Salford Royal NHS Foundation Trust, Manchester Academic Health Science Centre, University of Manchester, Greater Manchester, UK; 6 Institute of Psychiatry, Psychology and Neuroscience, King’s College London, London, UK; 7 Cognitive Neuroscience, Institute of Neuroscience and Medicine (INM-3), Research Centre Jülich, Jülich, Germany; 8 Department of Neurosciences (DNS), Padova University, Padova, Italy; 9 Center for Networked Biomedical Research in Neurodegenerative Diseases (CIBERNED), Carlos III Institute of Health, Madrid, Spain; 10 Chulalongkorn Centre of Excellence for Parkinson’s Disease & Related Disorders, Department of Medicine, Faculty of Medicine, Chulalongkorn University and King Chulalongkorn Memorial Hospital, Thai Red Cross Society, Bangkok, Thailand; 11 Faculty of Medicine, Transilvania University of Brașov, Brașov, Romania; 12 Department of Neurology, Seoul National University College of Medicine, Seoul, South Korea; 13 King’s College London, Institute of Psychiatry, Psychology & Neuroscience, London, UK; 14 Nuclear Medicine and PET, Aarhus University Hospital, Aarhus, Denmark; 15 University of Lund, Faculty of Medicine, Lund, Sweden; 16 UCL Institute of Neurology, London, UK; 17 Division of Neurodegenerative Diseases, Department of Neurology, Dresden University of Technology, Dresden, Germany; 18 Department of Neurology, Dresden University of Technology, Dresden, Germany; 19 German Center for Neurodegenerative Diseases (DZNE), Research Site Dresden, Dresden, Germany; 20 Movement Disorders Clinic, National Institute of Neurology and Neurosurgery, Mexico City, Mexico; 21 Department of Psychiatry and Department of Neurology, University of Pennsylvania School of Medicine, Philadelphia, PA USA; 22 Parkinson’s Disease and Mental Illness Research, Education and Clinical Centers, Philadelphia Veterans Affairs Medical Center, Philadelphia, PA USA; 23 The Parkinson’s Disease and Movement Disorders Center, Department of Neurology, Mayo Clinic, Scottsdale, AZ USA; 24 Center for Neurodegenerative Diseases (CEMAND), Neuroscience Section, University of Salerno, Salerno, Italy; 25 Institute of Neuroscience, Newcastle University, Newcastle, UK; 26 Department of Nuclear Medicine and PET Centre, Aarhus University Hospital, Aarhus, Denmark; 27 Department of Medicine, University of Birmingham Institute of Cardiovascular Sciences, City Hospital, Birmingham, UK; 28 Reviva Pharmaceuticals, Inc, Santa Clara, CA USA; 29 Faculty of Medicine and Dentistry, University of Plymouth, Plymouth, UK; 30 FAS Center for Systems Biology, Harvard University, Cambridge, MA USA; 31 Movement Disorder Clinic, University Hospital of Bispebjerg, Copenhagen, NV Denmark; 32 Oxford Parkinson’s Disease Centre, University of Oxford, Oxford, UK; 33 Nuffield Department of Clinical Neurosciences, University of Oxford, Oxford, UK; 34 Neurodegenerative Diseases Research Group, Institute of Pharmaceutical Sciences, Faculty of Life Sciences and Medicine, King’s College London, London, UK; 35 Division of Clinical Geriatrics, Department of Neurobiology, Care Sciences and Society, Center for Alzheimer Research, Karolinska Institutet, Stockholm, Sweden; 36 Department of Neurology, University Medical Centre Ljubljana, Ljubljana, Slovenia; 37 Parkinson’s Disease Service for the Older Person, Rankin Park Centre, John Hunter Hospital, HNELHD, Newcastle, NSW Australia; 38 Functional Movement Disorders Unit, Movement Disorders Program, Neurology Department, Hospital Ruber Internacional, Madrid, Spain; 39 Brain and Mind Centre, University of Sydney, Sydney, NSW Australia; 40 Department of Neurosciences Movement Disorders Center, University of California, San Diego, USA; 41 University of Kansas Medical Center, Kansas City, KS USA; 42 Department of Clinical Neurosciences, University of Calgary & Hotchkiss Brain Institute, Calgary, Canada; 43 Hurvitz Brain Sciences Program, Sunnybrook Research Institute, Toronto, ON Canada; 44 Department of Neurology, Osaka University Graduate School of Medicine, Osaka, Japan; 45 Parkinson Disease Research, Education, and Clinical Center, Philadelphia Veteran Affairs Medical Center, Philadelphia, PA USA; 46 Department of Neurology, University of Pennsylvania, Philadelphia, PA USA; 47 Department of Neurology, NYU School of Medicine, New York, NY USA; 48 Movement Disorders Unit, Sant Pau Hospital and Biomedical Research Institute (IIB-Sant Pau), Barcelona, Spain; 49 Neurology, National Hospital for Neurology & Neurosurgery, London, UK; 50 Newcastle Magnetic Resonance Centre & Positron Emission Tomography Centre, Newcastle University, Campus for Ageing & Vitality, Newcastle upon Tyne, UK; 51 Department of Neurology, Helsinki University Hospital, and Department of Neurological Sciences (Neurology), University of Helsinki, Helsinki, Finland; 52 Research Institute of McGill University Health Centre, Montréal, Canada; 53 Department of Neurology and Psychiatry, University of Santo Tomas Hospital, Manila, Philippines; 54 International Institute of Neuroscience, Saint Luke’s Medical Center, Manila, Philippines; 55 Center for Neurodiagnostic and Therapeutic Services, Metropolitan Medical Center, Manila, Philippines; 56 Department of Clinical Neurosciences, University College London (UCL) Institute of Neurology, Royal Free Campus, Rowland Hill Street, London, UK; 57 Department of Neurology, Northwestern University, Feinberg School of Medicine, Chicago, IL USA; 58 University and Institute for Research and Medical Care, IRCCS San Raffaele, Rome, Italy; 59 UCLA/West LA VA, Los Angeles, CA USA; 60 Cedars-Sinai Medical Center, Los Angeles, CA USA; 61 Department of Neuroscience, Biomedicine, and Movement, University of Verona, Verona, Italy; 62 Department of Pathology & Laboratory Medicine, University of Pennsylvania, Philadelphia, PA USA; 63 Department of Neurology, Houston Methodist Hospital, Houston, TX USA; 64 Department of Neurology, Fukuoka University, Fukuoka, Japan; 65 Northumbria Healthcare NHS Foundation Trust, North Tyneside General Hospital, Rake Lane, North Shields, Tyne and Wear UK

**Keywords:** Parkinson's disease, Quality of life

## Abstract

To identify predictors of 36-month follow-up quality of life (QoL) outcome after bilateral subthalamic nucleus deep brain stimulation (STN-DBS) in Parkinson’s disease (PD). In this ongoing, prospective, multicenter international study (Cologne, Manchester, London) including 73 patients undergoing STN-DBS, we assessed the following scales preoperatively and at 6-month and 36-month follow-up: PD Questionnaire-8 (PDQ-8), NMSScale (NMSS), Scales for Outcomes in PD (SCOPA)-motor examination, -activities of daily living, and -complications, and levodopa equivalent daily dose (LEDD). We analyzed factors associated with QoL improvement at 36-month follow-up based on (1) correlations between baseline test scores and QoL improvement, (2) step-wise linear regressions with baseline test scores as independent and QoL improvement as dependent variables, (3) logistic regressions and receiver operating characteristic curves using a dichotomized variable “QoL responders”/“non-responders”. At both follow-ups, NMSS total score, SCOPA-motor examination, and -complications improved and LEDD was reduced significantly. PDQ-8 improved at 6-month follow-up with subsequent decrements in gains at 36-month follow-up when 61.6% of patients were categorized as “QoL non-responders”. Correlations, linear, and logistic regression analyses found greater PDQ-8 improvements in patients with younger age, worse PDQ-8, and worse specific NMS at baseline, such as ‘difficulties experiencing pleasure’ and ‘problems sustaining concentration’. Baseline SCOPA scores were not associated with PDQ-8 changes. Our results provide evidence that 36-month QoL changes depend on baseline neuropsychological and neuropsychiatric non-motor symptoms burden. These findings highlight the need for an assessment of a wide range of non-motor and motor symptoms when advising and selecting individuals for DBS therapy.

## Introduction

Deep brain stimulation (DBS) of the subthalamic nucleus (STN) is a well-established therapy with long-term efficacy improving motor symptoms, quality of life (QoL), and non-motor symptoms (NMS) in patients with Parkinson’s disease (PD)^1–5^. Previous research also demonstrated beneficial effects of STN-DBS on QoL compared to medical treatment^6–8^. However, on the individual level, 43–49% of patients experience no clinically relevant improvement of QoL postoperatively at 6-month follow-up^6,9,10^. Furthermore, there is Class I evidence that in 36% of pairs of patients treated either with best medical treatment alone or with STN-DBS, medical treatment alone results in better QoL outcomes than STN-DBS^2^. Identifying preoperative factors that predict QoL outcome could support the decision-making process for DBS eligibility and improve individual treatment results. Amongst other parameters younger age, worse baseline QoL, and specific NMS have been identified as predictors of more considerable QoL improvement at 6-month follow-up. However, it is unclear which demographic and clinical parameters influence the evolution of QoL beyond such a short-term follow-up. Therefore, we investigated predictors of QoL outcome after STN-DBS at 36-month follow-up and, based on previous studies with shorter follow-up periods, hypothesized that QoL outcome depends on demographic and non-motor predictors as well as baseline QoL.

## Results

**Fig. 1:**
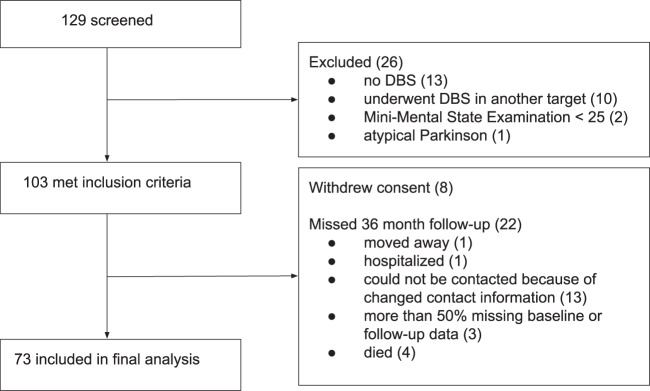
Enrollment. The flow chart describes the enrollment of patients. **DBS** deep brain stimulation.

Of 129 patients screened, 73 patients (43 male) were included in the final analysis (see Fig. 1). The mean age at baseline was 62.0 years (SD = 8.3) and disease duration 10.3 years (SD = 4.7). The mean time to follow-up was 3.0 years (SD = 0.31).

### Clinical outcomes at baseline, 6-month, and 36-month follow-up

Friedman tests revealed significant differences between the three visits for all outcome scores (see Table 1). In post hoc tests comparing baseline and 36-month follow-up, we observed significant longitudinal changes for the NMSS total score (*P* = 0.037), SCOPA-motor examination (*P* = 0.001), and -motor complications (*P* < 0.001), and significant sustained levodopa equivalent daily dose (LEDD) reduction (*P* < 0.001). No significant changes at 36-month follow-up were found for the PDQ-8 SI (*P* = 0.296) and SCOPA-activities of daily living (*P* = 0.161). PDQ-8 domains are reported in supplementary Table e-1.

**Table 1:** Outcome parameters at preoperative baseline and postoperative 6-month and 36-month follow-up.

	Baseline			6-month follow-up			36-month follow-up				
	*n*	*M*	SD	*n*	*M*	SD	*n*	*M*	SD	*p*^a^	Post hoc tests
PDQ-8 Summary Index	73	32.8	16.8	69	23.6	14.9	73	31.1	20.2	<0.001	a
NMSS-total score	73	57.3	34.9	69	39.1	24.6	73	47.6	31.4	<0.001	a b
SCOPA											
Motor examination	73	11.3	5.5	66	8.1	5.0	64	8.7	4.8	<0.001	a b
Activities of daily living	73	7.2	3.4	69	5.3	2.9	72	6.7	3.6	<0.001	a
Motor complications	73	4.9	3.0	69	2.2	2.5	71	2.3	2.4	<0.001	a b
LEDD	73	1146.2	508.2	69	550.1	339.0	72	722.7	469.4	<0.001	a b
MMSE	73	29.0	1.1	69	28.9	3.7	65	28.9	1.4	0.128	

*LEDD* levodopa equivalent daily dose, *MMSE* Mini-Mental State Examination, *NMSS* Non-Motor Symptom Scale, *PDQ-8* 8-item Parkinson’s Disease Questionnaire, *SCOPA* Scales for Outcomes in Parkinson’s Disease.

^a^Friedman test or repeated-measures ANOVA when parametric test criteria were fulfilled.

Benjamini-Hochberg correction was applied to account for multiple testing and all presented *P*-values were adjusted to the significance threshold *P* < 0.05. Baseline assessments were conducted in the medication ON state (MedON) and postoperative assessments in the medication ON/stimulation ON state (MedON/StimON).

Post hoc comparisons (Wilcoxon signed rank or *t* test):

a = significant difference between baseline vs 6-month follow-up (*P* < 0.05)

b = significant difference between baseline vs 36-month follow-up (*P* < 0.05)

### Correlation analyses

Table 2 shows correlations between PDQ-8 SI change score (baseline vs. 36-month follow-up) and demographic variables and preoperative clinical scores. Significant correlations were found between PDQ-8 SI changes and PDQ-8 SI_baseline_ (moderate strength) and age_baseline_ (weak). Correlations between improvement in PDQ-8 SI and NMSS total_baseline_ trended. Explorative Spearman correlations between PDQ-8 SI changes at 36-month follow-up and NMSS items at baseline showed significant associations with the items “difficulty experiencing pleasure” (NMSS-12_baseline,_ *r* = 0.24, *P* = 0.041), “concentration” (NMSS-16_baseline_, *r* = 0.34, *P* = 0.003), and “urinary frequency” (NMSS 23_baseline_, *r* = 0.27, *P* = 0.022). We observed no significant correlation between these NMSS items at baseline. A partial correlation between PDQ-8 SI change score and NMSS-16_baseline_ was still significant after controlling for NMSS-12_baseline_ (*r* = 0.31, *P* = 0.007).

**Table 2:** Correlations between preoperative baseline test scores or demographic variables and 36-month change scores of quality of life.

	PDQ-8 SI change score		
	*n*	*r*	*P*
Age	73	−0.29*	0.012
Sex^a^	73	−0.14	0.215
Disease duration	73	−0.02	0.860
PDQ-8 Summary Index	73	0.42***	<0.001
NMSS-total score	73	0.20	0.083
SCOPA			
Motor examination	73	0.01	0.938
Activities of daily living	73	0.14	0.233
Motor complications	73	−0.04	0.751
LEDD	73	0.04	0.713
MMSE	73	−0.06	0.599

Spearman correlations, respectively Pearson correlations for normally distributed variables were calculated between PDQ-8 SI change scores from preoperative baseline to postoperative 36-month follow-up and preoperative baseline test scores, respectively for demographic variables.

*LEDD* Levodopa equivalent daily dose, *MMSE* Mini-Mental State Examination, *NMSS* Non-motor Symptom Scale, *PDQ-8* 8-item Parkinson’s Disease Questionnaire, *SCOPA* Scales for Outcomes in Parkinson’s Disease.

*P < 0.050, **P < 0.010, ***P < 0.001.

^a^Rank-biserial correlation.

### Linear regression analysis

Univariate regression analyses were performed using the variables with a *P* < 0.2 in the correlation analyses as candidate predictors. This additionally included the items “fainting” (NMSS-2_baseline,_ *r* = −0.17, *P* = 0.157), “hallucinations” (NMSS-13_baseline,_ *r* = 0.18, *P* = 0.131), “forget things or events” (NMSS-17_baseline,_ *r* = 0.17, *P* = 0.157), “interest in sex” (NMSS-25_baseline,_ *r* = 0.21, *P* = 0.082), and “pain” (NMSS-27_baseline,_ *r* = 0.22, *P* = 0.063). Univariate regression analyses with change in PDQ-8 SI at 36-month follow-up as the criterion variable was significant for the following independent variables: PDQ-8 SI_baseline_ (*β* = 0.42, *P* < 0.001), age_baseline_ (*β* = –0.29, *P* = 0.012), NMSS total_baseline_ (*β* = 0.26, *P* = 0.025), NMSS-2_baseline_ (*β* = −0.25, *P* = 0.034), NMSS-12_baseline_ (*β* = 0.48, *P* < 0.001), NMSS-16_baseline_ (*β* = 0.37, *P* = 0.001), and NMSS 23_baseline_ (*β* = 0.25, *P* = 0.032). For the multivariate regression analysis, we excluded the variable NMSS total_baseline_ due to high intercorrelation with PDQ-8 SI_baseline_ (*r* = 0.65, *P* = <0.001). In the stepwise multivariate regression analysis, the variables age_baseline_, NMSS item 2_baseline_, NMSS item 12_baseline_, and NMSS item 16_baseline_ remained significant. The multivariate model accounted for 36% of the variance (*R*^2^ = 0.40) in PDQ-8 SI change (*F*_4,68_ = 11.1, *P* < 0.001). In this model, NMSS item 12_baseline_ had the highest predictive value (*β* = 0.35, *P* = 0.001), followed by age_baseline_ (*β* = −0.28, *P* = 0.004), NMSS item 16_baseline_ (*β* = 0.26, *P* = 0.011), and NMSS item 2_baseline_ (*β* = −0.22, *P* = 0.025).

### Logistic regression analysis

The cut-off for a clinically relevant change in PDQ-8 SI at 36-month follow-up was 8.4 points ($\frac{1}{2}$ SD of PDQ-8 SI_baseline_). Out of 73 patients in our cohort, 28 patients (38.4%) were classified as 36-month QoL “responders”, 29 patients (39.7%) reported unchanged QoL, and 16 patients (21.9%) indicated a clinically relevant worsening of long-term QoL.

For binary logistic regression analyses, patients reporting unchanged and worsened QoL were grouped as QoL “non-responders” (*n* = 45, 61.6%). In explorative logistic regression analyses, every additional year of age at baseline decreased the odds of 36-month QoL improvement by ~5% (odds ratio [OR]= 0.949, confidence interval [CI]=0.895–1.007, *P* = 0.082). Furthermore, the odds of QoL improvement were increased by ~5% with every additional point of baseline QoL impairment in the PDQ-8 SI_baseline_ (OR = 1.048, CI = 1.012–1.086, *P* = 0.008) and by ~14% for every additional 10 points of baseline non-motor burden in the NMSS-total score_baseline_ (OR = 1.014, CI = 0.999–1.029, *P* = 0.074). Moreover, specific NMSS items had a predictive value: one additional point in NMSS-12_baseline_ (“difficulties experiencing pleasure”) increased the odds of QoL improvement by 46% (OR = 1.462, CI = 1.054–2.028, *P* = 0.023) and in NMSS-16_baseline_ (“concentration”) by 30% (OR = 1.302, CI = 1.064–1.593, *P* = 0.010).

**Fig. 2:**
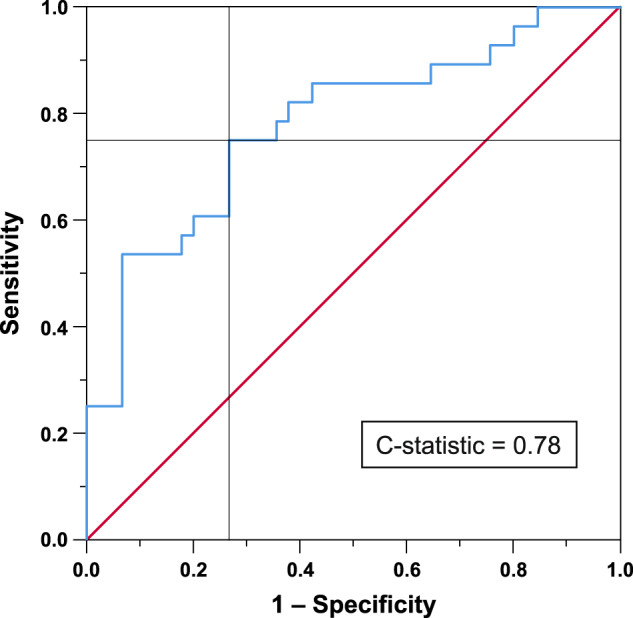
Receiver operating characteristic curve. The receiver operating characteristic curve (blue) illustrates the classification accuracy of the fitted logistic regression model (dependent variable: PDQ-8 SI “Responder”/”Non-Responder”, independent variables: age_baseline_, PDQ-8 SI_baseline_, NMSS-total score_baseline,_ NMSS item 12_baseline_, NMSS item 16_baseline_). The discriminatory power of the test with these parameters is demonstrated by C-statistic = 0.78. The diagonal line (red) represents chance classification accuracy. The cross of black reference lines indicates the optimal trade-off point in which the model reached 75.0% sensitivity and 73.3% specificity. NMSS Non-Motor Symptom Scale, PDQ-8 *SI*8-item Parkinson’s Disease Questionnaire Summary Index.

A logistic regression model using the aforementioned parameters (age_baseline_, PDQ-8 SI_baseline_, NMSS-total_baseline_, NMSS-12_baseline_, and NMSS-16_baseline_) correctly classified 75.3% of patients into groups of long-term QoL “responders/non-responders” (Nagelkerke’s *R*^2^ = 0.338, *χ*^2^ = 0.9, *P* = 0.001, *n* = 73) as opposed to only 61.6% without predictors. The model reached 75.0% sensitivity and 73.3% specificity at the optimal trade-off point (C-statistic = 0.779, *P* < 0.001, CI = 0.667–0.892, see Fig. 2)^11^.

Linear and logistic regression analyses were confirmed by Mann–Whitney *U* tests comparing baseline parameters between long-term QoL responders and non-responders. Significant differences were found for PDQ-8 SI (responders: 40.1 ± 20.4, non-responders 28.3 ± 12.5, *P* = 0.021), NMSS-12_baseline_ (responders: 1.7 ± 3.0, non-responders 0.4 ± 1.0, *P* = 0.015)_,_ NMSS-16_baseline_ (responders: 3.1 ± 2.9, non-responders 1.5 ± 2.1, *P* = 0.009), NMSS-23_baseline_ (responders: 3.1 ± 4.0, non-responders 1.6 ± 3.0, *P* = 0.017), and NMSS-27_baseline_ (responders: 3.5 ± 3.7, non-responders 2.0 ± 3.5, *P* = 0.027).

## Discussion

In the present study, we report the 36-month effects of STN-DBS on QoL in a cohort of 73 patients with PD. We observed significant improvements in QoL following STN-DBS at a short-term, i.e., 6-month follow-up with subsequent decrements in gains at 36-month follow-up when only 38% of the patients experienced a sustained clinically relevant QoL improvement compared to preoperative baseline. Our results provide evidence that clinically relevant QoL improvement three years after preoperative baseline assessment can be predicted with 75% accuracy. Greater QoL improvement was observed for patients with younger age at intervention, worse baseline QoL, and a higher burden of specific NMS, such as anhedonia and concentration impairments. In contrast, patients more severely affected by fainting at baseline experienced less QoL improvement.

To our knowledge, the present study is the first to report an association between younger age at intervention and greater QoL improvement at 36-month follow-up. The association between these parameters was previously described for a 12-month period by Soulas et al.^12^. However, other studies found no association between age and changes in QoL^6,13^. This inconsistency might be explained by the fact that calendar age may not predict QoL. Instead, QoL after STN-DBS may be associated with ‘physiological age’. For example, frailty and co-morbidities may impact QoL post STN-DBS more than calendar age^14^. In line with previous research, other sociodemographic parameters, such as sex and disease duration were not significantly correlated with long-term change of QoL^6,15^.

Confirming results of earlier studies with shorter follow-up periods, the dosage of dopaminergic medication at preoperative baseline was not associated with QoL outcome^6,13^. In line with previous studies with follow-up periods up to 5 years, motor examination did not predict QoL changes^13,16^. In line with previous studies with follow-up periods up to 5 years, motor examinations did not predict QoL changes. Daniels et al.^6^ reported that the cumulative daily OFF time is the strongest predictor for improvement in disease-related QoL after DBS at 6-month follow-up. Further studies including cumulative OFF time with a longer follow-up are needed.

To our knowledge, this is the first report of a significant relationship between more severe preoperative QoL impairment and greater postoperative QoL improvement at 36-month follow-up^3^. This is in line with previous studies, that reported a relationship between these parameters at 6-month and 24-month follow-up^3,9^. Every additional point in the PDQ-8 SI at baseline increased the odds of favorable long-term QoL outcome by 5%. The strength of the association is in line with the results of our previous study at short-term follow-up^9^ and the Cleveland Clinic cohort results^10^, emphasizing the essential role of baseline QoL for the prediction of even long-term QoL outcome and also demonstrating the validity of our results. Our results are in line with several previous studies which have demonstrated that higher baseline QoL impairments predict greater postoperative QoL improvement at short-term follow-up^3,6,10^. In contrast, a study by Lezcano et al.^16^ has observed that lower less severe QoL impairments could predict greater QoL improvement at 1- and 5-year follow-up. These differences could be explained by demographic and clinical parameters in the study by Lezcano et al., such as a longer mean disease duration (13.2 years) and higher mean baseline PDQ impairments (41.1 points), than in the present study (10.3 years and 32.8 points)^3^^,6,10,16^. In the multivariate model, anhedonia, age, concentration problems and fainting contributed toward explaining QoL outcome at 36-month follow-up, whereas baseline QoL did not add to the predictive value of this model. This means that, although baseline QoL was a significant predictor of QoL change at 36-month follow-up in the univariate analysis, its contribution in the multivariate model was dominated by the other four variables mentioned earlier.

In the present study, specific preoperative NMS, namely more severe anhedonia and problems with sustaining concentration, were predictors for greater QoL improvement.

The predictive potential of *depressive symptoms* is in line with the results at 6-month follow-up in a previous study of our group^9^ and 8-month follow-up in the Cleveland Clinic cohort^10^. The present study results also extend the time frame of a 24-month follow-up study by Schuepbach et al. which reported greater QoL improvement in patients with worse baseline scores in two depression scales (Beck Depression Inventory and Montgomery-Åsberg Depression Rating Scale)^3^. One must acknowledge, that preoperative psychological interviews and strict formal testing resulted in a highly selected cohort with low baseline depression similar to other cohorts^2,3,17^. Therefore, the observation that worse baseline depression results in greater QoL improvements is only valid for patients with minimal or subclinical depression. More severe preoperative depression is a known risk factor for postoperative attempted or completed suicide^18^.

Furthermore, we observed that patients with greater baseline *concentration* deficits experienced greater QoL improvements at 36-month follow-up. The relationship between baseline concentration and QoL changes remained significant after controlling for anhedonia. Floden et al. and Witt et al. have reported that higher preoperative verbal memory deficits (Rey Auditory Verbal Learning Test single-trial memory and Dementia Rating Scale-2) are predictors of more unsatisfactory postoperative QoL outcome at 6- and 8-month follow-up^10,19^. Concentration/attention deficits are often accompanied by global cognition impairment in patients with PD. However, in our cohort, multi-disciplinary team assessments included expert neuropsychological assessments with formal testing of global cognition scores, psychiatric interviews, and neurological examinations to identify risks of adverse outcomes in patients with poor preoperative global cognition as these patients have a higher risk to progress to dementia. Strict indication assessments resulted in normal global cognition at baseline which remained stable at 6- and 36-month follow-up. Therefore, in this highly selected cohort, a higher burden of isolated concentration deficits constituted a predictor of greater QoL improvement. Future studies in larger cohorts including formal testing of concentration are warranted to confirm this finding.

To our knowledge, our study is the first to report an association between the presence of preoperative fainting and worse QoL outcome at 36-month follow-up. This finding is in line with the observation that cardiovascular symptoms, such as fainting/syncopes, worsen at 36-month follow-up^8^ and have a marked negative impact on QoL^20^.

Some limitations of our study should be acknowledged. One important limiting factor is the underrepresentation of patients with severe NMS, such as clinically relevant psychiatric disorders or cognitive impairment, as these patients were not eligible for DBS. Although the cohort size of the present study (*n* = 73) is limited, it is still one of the largest beyond short-term follow-up. Furthermore, the multicenter design of our study increases external validity by reducing bias caused by single-center studies. We did not systematically assess apathy, which could have improved our prediction model, as patients with negative QoL outcome showed higher preoperative apathy scores in previous research^21^. QoL was assessed with the PDQ-8, which may be less sensitive to small QoL changes than the PDQ-39 due to a reduced scale gradation resulting from fewer items^22^. Due to the focus on QoL and non-motor aspects of PD, we did not conduct assessments of motor examination in pre- or postoperative medication or stimulation OFF states and we did not assess other motor aspects, such as the cumulative daily OFF time or severity of dyskinesia. Future studies are needed to further explore a possible predictive potential of these parameters. Another limitation is that severe disease progression can result in patients being lost to follow-up which could introduce a systematic bias in studies with longer follow-up periods^23^.

Also, the variability of the exact location of stimulation in the target area might be relevant for postoperative QoL improvement^24^, but was not investigated in the present study as we focused on preoperative predictors of QoL outcome. A recent study by Petry-Schmelzer et al. reported that non-motor outcomes, such as mood/apathy and attention/memory, depend on the location of neurostimulation and are correlated with QoL outcome^24–26^. These results and the predictive value of baseline anhedonia and concentration deficits observed in the present study highlight the importance of assessments of a wide range of NMS which may have implications for DBS programming to achieve optimal long-term QoL outcomes.

The observation of greater QoL improvements at 36-month follow-up in patients with younger age at intervention, worse preoperative QoL, worse preoperative anhedonia and concentration problems, and less autonomic dysfunction, such as fainting, highlight the importance of preoperative assessments of a wide range of motor and nonmotor symptoms. Our results, therefore, contribute to the long-term goal of identifying patients who experience more considerable postoperative QoL improvement and optimizing patient selection for STN-DBS.

## Methods

### Study design

In this ongoing, prospective, observational, multicenter international study (Cologne, London, Manchester), we examined patients with PD undergoing STN-DBS as part of the DBS arm of the NILS study at preoperative baseline, 6-month, and 36-month follow-up postoperatively^27^^,28^. Patients were screened between 06/2011 and 07/2017. The study was conducted under the Declaration of Helsinki. Study protocols had been approved by the local ethics committees (Cologne, study no.: 12-145; German Clinical Trials Register: DRKS00006735; United Kingdom: NIHR portfolio, number: 10084; National Research Ethics Service South East London REC 3, 10/H0808/141). All patients gave written informed consent before study procedures.

### Participants

PD diagnosis was based on the UK Brain Bank criteria and patients were screened for DBS treatment according to the guidelines of the International PD and Movement Disorders Society^29^. A sufficient levodopa responsiveness (>30% improvement in the Unified Parkinson’s Disease Rating Scale-III) was required for each patient. Furthermore, eligibility for STN-DBS was based on multi-disciplinary assessments including movement disorders specialists, stereotactic neurosurgeons, neuropsychologists, psychiatrists, and when necessary, speech therapists and physiotherapists. This led to the exclusion of patients with clinically relevant cognitive impairment and psychiatric diseases^30^.

### Clinical assessment

Clinical assessments were carried out under medication ON (MedON) at preoperative baseline and with neurostimulation ON and medication ON (MedON/StimON) at 6-month and 36-month follow-up.

The following scales and questionnaires were assessed:

1. QoL was investigated with the PD Questionnaire-8 (PDQ-8) reported as PDQ-8 Summary Index (PDQ-8 SI) ranging from 0 (no impairment) to 100 (maximum impairment)^31,32^. The PDQ-8 assesses eight aspects of QoL (mobility, activities of daily living, emotional wellbeing, stigma, social support, cognition, communication, bodily discomfort) and has been commonly used in PD^33^ and STN-DBS^28,34,35^.
2. The clinician-rated NMS Scale (NMSS) contains 30 items covering nine domains of NMS: cardiovascular, sleep/fatigue, mood/apathy, perceptual problems/hallucinations, attention/memory, gastrointestinal tract, urinary, sexual function, and miscellaneous (including pain, inability to smell/taste, weight changes, and sweating). Symptoms are surveyed over the last four weeks and therefore reflect ON and OFF states. The NMSS total score ranges from 0 (no impairment) to 360 (maximum impairment)^36^.
3. Motor examination, activities of daily living, and motor complications were assessed with the Scales for Outcomes in PD (SCOPA) -motor examination, -activities of daily living, and -motor complications^37^. The SCOPA is an abbreviated version of the Unified PD Rating Scale. It strongly correlates with the corresponding parts of the Unified PD Rating Scale and was used here as its administration time is approximately four times shorter than the MDS-Unified PD Rating Scale^37,38^. SCOPA subscales range from 0 (no impairment) to 42 (motor examination), 21 (activities of daily living), and 12 (motor complications). Motor examinations were conducted by movement disorders specialists.
4. Global cognition was assessed with the Mini-Mental State Examination (MMSE) which ranges between 0 (maximum impairment) and 30 (no impairment).
5. To record the medical regimen, we calculated the LEDD following Tomlinson et al.^39^.

### Statistical analysis

#### Longitudinal outcome changes

Statistical analyses were performed using SPSS Statistics 26. The Kolmogorov-Smirnov test was applied to check the assumption of normality. Longitudinal outcome changes between the three visits were analyzed with Friedman tests or repeated-measures analyses of variance when parametric test criteria were fulfilled. Post hoc, we calculated Wilcoxon signed-rank and *t*-tests, respectively, to compare outcome changes between pairs of visits. Benjamini-Hochberg correction was applied to account for multiple testing. The presented *P*-values were adjusted to the significance threshold *P* < 0.05 unless stated otherwise.

#### Correlation analyses

The relationship between changes in QoL scores and preoperative demographic and clinical parameters was explored using Spearman correlations, respectively Pearson correlations for normally distributed variables. PDQ-8 SI change score (mean Test_baseline_ – mean Test_36-month follow-up_) was correlated with the following variables: age_baseline_, sex, disease duration since diagnosis, NMSS total score_baseline_, PDQ-8 SI_baseline_, SCOPA-motor examination_baseline_, -activities of daily living_baseline_, -motor complications_baseline_, MMSE_baseline_, and LEDD_baseline_. In addition, we explored if PDQ-8 SI change score correlated to specific NMSS items_baseline_ and, when appropriate, if these results remained significant after controlling for changes in other NMSS items in partial correlations.

#### Linear regression analysis

In a second step, we aimed to identify preoperative predictors of long-term QoL outcome using stepwise linear regression analysis. We included parameters from the correlation analyses (*P* < 0.2)^40^ as candidate predictor variables and PDQ-8 SI change score as criterion variable. Multi-collinearity was checked using intercorrelations between candidate predictor variables (*r* > 0.6) and Variance Inflation Factors, which should not exceed 10^41^.

#### Logistic regression analyses and receiver operating characteristics

Furthermore, the cohort was divided into groups of patients with clinically relevant QoL improvement and patients reporting stable/worsened QoL at 36 months. Each patient was classified as a long-term QoL “responder” or “non-responder” based on a preassigned threshold (½ SD of PDQ-8 SIbaseline) to report clinically important differences^42^. We employed exploratory logistic regression models and receiver operating characteristic analyses with dichotomized QoL outcome as criterion variable and demographic and preoperative clinical parameters as predictor variables to evaluate the utility of linear regression models to predict patients’ postoperative long-term QoL changes. Moreover, we analyzed differences of baseline characteristics between “responders”/“non-responders” using Mann–Whitney *U* tests or *t*-tests, respectively. To explore the relationship between QoL outcome changes and specific NMS, all analyses were explored for NMSS item scores.

### Reporting summary

Further information on research design is available in the Nature Research Reporting Summary linked to this article.

## Supplementary information

Reporting Summary

Supplementary Information

## Data Availability

The data included in this study are available on request to the corresponding author. The data are not publicly available due to their containing information that could compromise the privacy of the participants.

## Code availability

Statistical analyses were performed using SPSS Statistics 26. SPSS codes are available upon reasonable request to the corresponding authors.
