# Supplementary material for: Non-motor predictors of 36-month quality of life after subthalamic stimulation in Parkinson disease
Source: NPJ Parkinsons Dis. 2021 Jun 8;7:48. doi: 10.1038/s41531-021-00174-x (PMC8187358; doi:10.1038/s41531-021-00174-x)
Supplement: Supplementary file 1 — Reporting Summary [file 41531_2021_174_MOESM1_ESM.pdf]

nature research

Corresponding author(s): Dr. Haidar S. Dafsari and Stefanie T. Jost

Last updated by author(s): Nov 19, 2020

Reporting Summary

Nature Research wishes to improve the reproducibility of the work that we publish. This form provides structure for consistency and transparency in reporting. For further information on Nature Research policies, see our Editorial Policies and the Editorial Policy Checklist.

Statistics

For all statistical analyses, confirm that the following items are present in the figure legend, table legend, main text, or Methods section.

n/a	Confirmed	
☐	☒	The exact sample size (n) for each experimental group/condition, given as a discrete number and unit of measurement
☐	☒	A statement on whether measurements were taken from distinct samples or whether the same sample was measured repeatedly
☐	☒	The statistical test(s) used AND whether they are one- or two-sided<br>*Only common tests should be described solely by name; describe more complex techniques in the Methods section.*
☐	☒	A description of all covariates tested
☐	☒	A description of any assumptions or corrections, such as tests of normality and adjustment for multiple comparisons
☐	☒	A full description of the statistical parameters including central tendency (e.g. means) or other basic estimates (e.g. regression coefficient) AND variation (e.g. standard deviation) or associated estimates of uncertainty (e.g. confidence intervals)
☐	☒	For null hypothesis testing, the test statistic (e.g. F, t, r) with confidence intervals, effect sizes, degrees of freedom and P value noted<br>*Give P values as exact values whenever suitable.*
☐	☒	For Bayesian analysis, information on the choice of priors and Markov chain Monte Carlo settings
☐	☒	For hierarchical and complex designs, identification of the appropriate level for tests and full reporting of outcomes
☐	☒	Estimates of effect sizes (e.g. Cohen's d, Pearson's r), indicating how they were calculated

Our web collection on statistics for biologists contains articles on many of the points above.

Software and code

Policy information about availability of computer code

Data collection	No software was used.
Data analysis	SPSS V.26.0 (IBM Corporation)

For manuscripts utilizing custom algorithms or software that are central to the research but not yet described in published literature, software must be made available to editors and reviewers. We strongly encourage code deposition in a community repository (e.g. GitHub). See the Nature Research guidelines for submitting code & software for further information.

Data

Policy information about availability of data

All manuscripts must include a data availability statement. This statement should provide the following information, where applicable:
- Accession codes, unique identifiers, or web links for publicly available datasets
- A list of figures that have associated raw data
- A description of any restrictions on data availability

Data are available upon reasonable request. The data included in this study are available on request to the corresponding author. The data are not publicly available due to their containing information that could compromise the privacy of the participants.

Field-specific reporting

Life sciences study design

All studies must disclose on these points even when the disclosure is negative.

Sample size	No sample-size calculation was performed as this was a registry study.
Data exclusions	No data was excluded from the analyses
Replication	Not applicable as this is a registry study.
Randomization	A randomised controlled design was not suitable as in this study, investigations of long-term effects would otherwise have resulted in withholding an effective therapy from severely affected patients for 3 years
Blinding	As the present work analyzed data from an observational study, we did not conduct blinded assessments of clinical outcomes

Reporting for specific materials, systems and methods

We require information from authors about some types of materials, experimental systems and methods used in many studies. Here, indicate whether each material, system or method listed is relevant to your study. If you are not sure if a list item applies to your research, read the appropriate section before selecting a response.

Materials & experimental systems

n/a	Involved in the study	
☒	☐	Antibodies
☒	☐	Eukaryotic cell lines
☒	☐	Palaeontology and archaeology
☒	☐	Animals and other organisms
☐	☒	Human research participants
☐	☒	Clinical data
☒	☐	Dual use research of concern

Methods

n/a	Involved in the study	
☒	☐	ChIP-seq
☒	☐	Flow cytometry
☒	☐	MRI-based neuroimaging

Human research participants

Policy information about studies involving human research participants

Population characteristics	The mean age at baseline was 62.0 years (SD=8.3) and disease duration 10.3 years (SD=4.7). PD diagnosis was based on the UK Brain Bank criteria and patients were screened for DBS treatment according to the guidelines of the International PD and Movement Disorders Society.
Recruitment	This was a multicenter international registry study (Cologne, London, Manchester). Patients with PD undergoing STN-DBS were recruited and examined as part of the DBS arm of the NILS study.
Ethics oversight	Study protocols had been approved by the local ethics committees (Cologne, study no.: 12-145; German Clinical Trials Register: DRKS00006735; United Kingdom: NIHR portfolio, number: 10084; National Research Ethics Service South East London REC 3, 10/H0808/141).

Note that full information on the approval of the study protocol must also be provided in the manuscript.

Clinical data

Policy information about clinical studies

All manuscripts should comply with the ICMJEguidelines for publication of clinical research and a completedCONSORT checklist must be included with all submissions.

Clinical trial registration	German Clinical Trials Register: DRKS00006735
Study protocol	The study protocol is available upon reasonable request.
Data collection	In this ongoing, prospective, observational, multicenter international study (Cologne, London, Manchester), we examined patients with PD undergoing STN-DBS as part of the DBS arm of the NILS study at preoperative baseline, 6-month, and 36-month follow-up postoperatively. Patients were screened between 06/2011 and 07/2017.
Outcomes	The following scales and questionnaires were assessed:<br>1) QoL was investigated with the PD Questionnaire-8 (PDQ-8) reported as PDQ-8 Summary Index (PDQ-8 SI).<br>2) The clinician-rated NMS Scale (NMSS) contains 30 items covering nine domains of NMS.<br>3) Motor examination, activities of daily living, and motor complications were assessed with the Scales for Outcomes in PD (SCOPA) - motor examination, -activities of daily living, and -motor complications.

4) Global cognition was assessed with the Mini-Mental State Examination (MMSE) which ranges between 0 (maximum impairment) and 30 (no impairment).
5) To record the medical regimen, we calculated the levodopa equivalent daily dose.
