# Supplementary material for: Non-motor predictors of 36-month quality of life after subthalamic stimulation in Parkinson disease
Source: NPJ Parkinsons Dis. 2021 Jun 8;7:48. doi: 10.1038/s41531-021-00174-x (PMC8187358; doi:10.1038/s41531-021-00174-x)
Supplement: Supplementary file 2 — Supplementary Information [file 41531_2021_174_MOESM2_ESM.pdf]

Supplementary Table 1 – PDQ-8 domains at preoperative baseline and postoperative 6-month and 36-month follow-up

	Baseline			6-month follow-up			36-month follow-up				
	n	M	SD	n	M	SD	n	M	SD	p†	Post hoc tests
PDQ-8 Summary Index	73	32.8	16.8	69	23.6	14.9	73	31.1	20.2	<0.001	a
Mobility	73	1.8	1.1	69	1.3	1.1	73	1.5	1.2	0.005	a b
Activity of daily living	73	1.5	1.2	69	1.0	1.1	73	1.3	1.2	<0.001	a
Emotional well-being	73	1.1	1.0	69	0.8	0.9	73	1.2	1.1	0.001	a
Stigma	73	1.0	1.2	69	0.6	0.9	73	0.7	1.0	0.011	a b
Social support	73	1.0	1.0	69	0.9	0.9	73	1.1	1.0	0.110	
Cognitions	73	1.4	1.0	69	1.0	1.1	73	1.4	1.1	0.002	a
Communication	73	1.1	1.0	69	1.0	1.0	73	1.4	1.2	0.004	b
Bodily discomfort	73	1.6	1.2	69	1.1	1.1	73	1.4	1.2	0.002	a

Abbreviations: PDQ-8 = 8-item Parkinson's Disease Questionnaire

† Friedman test

Post hoc comparisons (Wilcoxon signed rank or t test):

a = significant difference between baseline vs 6-month follow-up (P<0.05)

b = significant difference between baseline vs 36-month follow-up (P<0.05)
